# The Medical Colleges of This City, Their Closing Exercises

**Published:** 1885-04

**Authors:** 


### THE MEDICAL COLLEGES OF THE CITY—THEIR CLOSING EXERCISES.

### THE ATLANTA MEDICAL COLLEGE.

The commencement exercises of this College were held in DeGive's Opera House, on March 2d, before a large and fashionable audience. On the stage were seated the Board of Trustees and the Faculty, with the speakers of the evening, Dr. J. B. Hawthorne and Col. Albert H. Cox.

After a report to the Trustees, by Dr. James A. Gray, the Proctor, the following gentlemen were called upon the stage and received the degree at the hands of H. H. Tucker, D. D.:

From Georgia—J. L. Baker, W. W. Binion, G. P. Brice, W. H. Leyden, T. D. McKown, F. W. McRae, J. B. Mitchell, M. T. Bramblett, J. D. Burt, T. K. Chapman, A. C. Clements, T. B. Cunningham, J. H. Morgan, R. W. Mürray, J. D. Pickett, D. J. Rogers, T. J. Dewberry, J. A. Dunwody, A. J. Fowler, I. A. Gibson, J. A. Guinn, S. W. Stiles, W. M. Terrell, C. I. Thompson, O. W. Tucker, J. G. Underwood, W. J. Hall, J. M. Hooten, S. F. Jowers, C. C. King, J. F. Wilson, T. A. White, W. F. Westmoreland, Jr.

From South Carolina—J. D. Austin, P. K. Black, W. F. Leonard, Van Smith, J. A. White.

From Alabama—A. J. Fowler, S. F. Jowers.

From North Carolina—J. H. Morgan.

Dr. Gray introduced Dr. A. C. Clements, the valedictorian, who delivered an address of half an hour's length. He spoke of the sadness of an occasion which was to mark the separation of old college friends, but while all regretted the parting, it was nevertheless an occasion for great rejoicing. It was but the beginning of the career of usefulness to which each member of the class had looked forward with so much hope and with such high anticipations. He warned his classmates that they would have cares and responsibilities, and urged them to be brave and bear them. It is only in trials that strength can be secured and true manhood developed. He referred to the great advances in science, urged the members of the class to be perfect gentlemen as well as good doctors, and thanked the people of Atlanta and the members of the college faculty for many kindnesses. His speech was listened to with close attention and ended amid applause.

Dr. J. B. Hawthorne was then introduced and delivered the annual address to the graduating class. [The address will be found published in full in another part of THE JOURNAL.]

After Dr. Hawthorne's address the next thing on the programme was the awarding of the prizes.

The Faculty offered three prizes, to be known as the first, second and third honor prizes, to be awarded to the three students who, at the examination for their degree, had attained the highest general proficiency in their studies. These prizes—handsome gold medals—were awarded to Dr. J. D. Austin, of South Carolina; Dr. F. W. McRae, of Georgia; and Dr. A. C. Clements, of Georgia. Honorable mention was also made of Dr. J. A. Dunwody.

The other prizes were awarded as follows:

Professor W. F. Westmoreland's prize of a transfusion apparatus to the student who presents the best report of surgical clinic, to Dr. J. A. Dunwody.

Professor Taliaferro's prize, an obstetrical forceps for the best report of his clinic, to Dr. A. C. Clements.

Professor Calhoun's prize of an ophthalmoscope, to the student who presents the best report of the eye and ear clinic, to Dr. J. A. Dunwody.

Professor William Abram Love's prize, a medical case, for the best essay on the nervous and vascular relations of the ovaries and uterus to the general system, to Dr. J. A. Dunwody.

Professor Logan's prize, a chemical test case, for the best thesis on the relations of the science of chemistry to medicine, to Dr. I. A. Gibson.

The quiz masters' prize was awarded to Dr. J. A. Dunwody.

These prizes were delivered by Hon. Albert Cox in an address that fairly captivated the audience.

The benediction was pronounced by Dr. Tucker.

The occasion was a most enjoyable one in every way.

### SOUTHERN MEDICAL COLLEGE.

The commencement exercises of the Southern Medical College took place at DeGive's Opera House, on the evening of March the 4th, 1885. There was a very large audience in attendance.

The exercises were opened with prayer by the Rev. Dr. Mack, of Columbia Theological Seminary, after which Dr. W. P. Nicolson, the Dean, made his Annual Report.

The following is the list of graduates: J. W. Anderson, Georgia; Oscar Attaway, Georgia; J. P. Ballenger, Georgia; E. V. Blount, North Carolina; R. R. Bomar, Georgia; G. E. Camp, Georgia; B. L. Clifton, Georgia; W. J. Davie, Kentucky; T. S. Few, South Carolina; E. M. Fowler, Georgia; H. W. Guthrie, Georgia; J. H. Hall, Georgia; Charles Hamilton, Georgia; P. E. Hannah, Florida; W. I. Hayes, Alabama; Jos. Hollingsworth, Georgia; D. B. Jackson, South Carolina; S. M. Kimsey, Georgia; Nathan King, Georgia; R. M. McBee, Tennessee; W. Y. McClure, Georgia; M. T. Maxwell, Georgia; C. A. Moran, Georgia; J. E. Rogers, North Carolina; W. P. Rushin, Georgia; W. B. Sanders, Alabama; J. W. Saunders, Georgia; G. W. Scroggs, Georgia; S. G. Smith, South Carolina; J. W. Suggs,

Jr., Georgia; J. O. Whatley, Georgia; H. C. Barnett, M. D., Texas, Ad Eundem Degree.

The valedictory address was made by Dr. J. W. Suggs, Jr., of Georgia, and the annual address was made by Dr. J. G. Armstrong, D. D., of this city. The first and second honor prizes were awarded to Dr. T. S. Few, of South Carolina, and to Dr. J. H. Hall, of Georgia. The faculty prizes were awarded as follows:

Eye, Ear and Throat, an ophthalmoscope to Dr. J. H. Hall, of Georgia, by Prof. A. G. Hobbs.

Practice, a gold medal to Dr. T. S. Few, of South Carolina, by Prof. W. D. Bizzell.

Materia Medica, a case of instruments to Dr. W. B. Sanders, of Alabama, by Prof. G. G. Roy.

Chemistry, a test apparatus to Dr. M. T. Maxwell, of Georgia, by Prof. J. A. Burns.

Physiology, a pocket drug case to Dr. W. P. Rushin, of Georgia, by Prof. R. C. Word.

Obstetrics, a gold medal to Dr. J. H. Hall, of Georgia, by Prof. T. S. Powell.

Surgery, a case of pocket instruments to Dr. J. H. Hall, of Georgia, by Prof. J. McF. Gaston.

Anatomy, a case of pocket instruments to Dr. T. S. Few, of South Carolina, by Dr. W. P. Nicolson.

